# The burden of gastroenteritis in Switzerland (BUGS) study: a research proposal for a 1-year, prospective cohort study

**DOI:** 10.1186/s13104-018-3916-2

**Published:** 2018-11-16

**Authors:** Claudia Schmutz, Daniel Mäusezahl

**Affiliations:** 1 Swiss Tropical and Public Health Institute, Basel, Switzerland; 2 University of Basel, Basel, Switzerland

**Keywords:** Research proposal, Cohort study, Acute gastroenteritis, Burden of disease, Incidence, Aetiology, Antibiotic resistance, Switzerland

## Abstract

**Objectives:**

Acute gastroenteritis (AG) is a usually self-limiting, but common disease worldwide. In Europe, incidence estimates range from 0.3–1.5 AG episodes/person-year. For Switzerland, available information on AG is restricted to notifiable foodborne diseases and findings from research studies starting at primary care level. The aims of this 1-year, population-based prospective cohort study are to assess the incidence, burden of disease, aetiology and socio-economic impact of AG in the Swiss general population. Additionally, the prevalence of bacterial gastrointestinal pathogens and bacteria harbouring antimicrobial resistances in the asymptomatic population shall be assessed.

**Results:**

Weekly follow-up of the cohort consisting of 3000 participants will provide incidence estimates of AG. Furthermore, information collected will be used to assess risk factors for experiencing an episode of AG, to explore determinants for help seeking, and to characterise the socio-economic impact of AG including absence from work and inability to perform daily activities. Aetiology of AG is determined by investigating stool samples from symptomatic participants. Finally, stool samples from participants collected during an asymptomatic period will be used to assess the prevalence of enterohaemorrhagic *E. coli*, *Campylobacter* spp., *Salmonella* spp. and *Shigella* spp. as well as of resistance to different antibiotics (extended-spectrum beta-lactamase-, fluoroquinolone- and carbapenemase-resistance).

**Electronic supplementary material:**

The online version of this article (10.1186/s13104-018-3916-2) contains supplementary material, which is available to authorized users.

## Introduction

Acute gastroenteritis (AG), manifesting with signs and symptoms of diarrhoea, vomiting, abdominal pain or cramps, fever, dehydration, nausea and/or loss of appetite, is usually self-limiting, but leads to a considerable burden of disease, health system use and socio-economic impact. Studies in several European countries estimated the incidence of AG at 0.3–1.5 episodes per person and year [1–14]. Furthermore, it was found that a considerable 11% of patients with infectious enteritis develop post-infectious irritable bowel syndrome [15, 16]. Incidence of AG for Switzerland is assumed to be comparable, but data is limited to notifiable pathogens reported to the Federal Office of Public Health (FOPH) based on the Epidemics Act. Several studies from other European countries have shown that (i) only 6.4–37.8% of all AG episodes lead to consultation of a physician [1–9, 11, 12, 14], and (ii) 0.2–1.8% of episodes are reported to national surveillance systems [1, 11, 14]. These proportions are highly variable between countries and pathogens due to different help seeking behaviour, case management and surveillance systems [17]. From a study in the Swiss Sentinel Surveillance Network, Sentinella, we estimated that around 175,000 individuals consulted a physician due to AG in Switzerland in 2014 [18]. Around 12% of cases were asked to submit a stool specimen and hence, could potentially—if positive—be reported to the National Notification System for Infectious Diseases (NNSID) if their sample tested positive for a notifiable pathogen and was reported by the diagnostic laboratory. However, the proportion of AG patients consulting a physician is currently not known for Switzerland. Consequently, inference from the above-mentioned frequencies and proportions to the incidence and burden of AG at population level is not possible.

Therefore, the present study primarily aims at measuring the incidence of acute gastroenteritis in the general population in Switzerland. Secondary objectives are to describe the burden of disease and the socio-economic impact of AG, to assess its aetiology and to investigate the frequency of selected risk exposures. Finally, the carriage rate of selected pathogenic bacteria and bacteria harbouring selected antibiotic resistances among the “healthy” (non-diarrhoeal) population in Switzerland is assessed.

## Main text

### Study design and methodology

A 1-year, prospective cohort study is conducted to determine the incidence of AG in the general population in Switzerland. The study will assess signs and symptoms of AG and exposure to selected risk factors (incl. antibiotic use). In case of symptoms, the help seeking behaviour of patients, inability to work or to perform usual daily activities, perceived illness experience and socio-economic consequences of the illness will be explored. Furthermore, the aetiology of AG is investigated by examining stool samples from a subsample of participants reporting gastrointestinal symptoms. Finally, the prevalence of selected bacterial gastrointestinal pathogens (enterohaemorrhagic *Escherichia coli* [EHEC], *Campylobacter*, *Salmonella* and *Shigella*) and bacteria with selected antibiotic resistances (extended-spectrum beta-lactamase [ESBL], carbapenemase, fluoroquinolone, and mobilised colistin resistance [mcr]-1) is assessed among participants during an asymptomatic period.

#### Study setting, recruitment process and eligibility

**Fig. 1:**
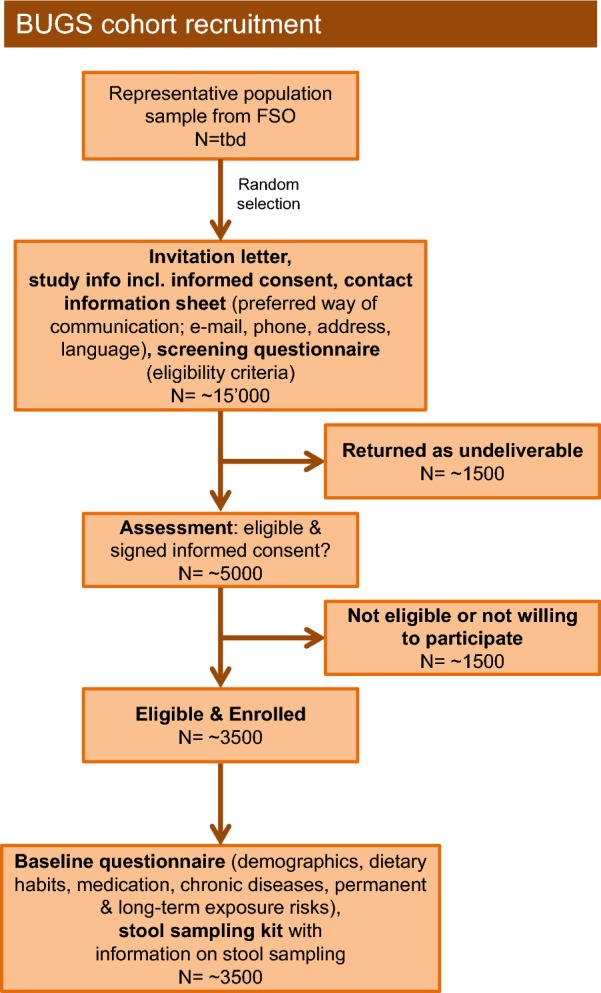
Operational flowchart of cohort recruitment for the burden of gastroenteritis in Switzerland (BUGS) study. FSO, Federal Statistical Office; tbd, to be defined

A representative sample of the Swiss population will be requested from the Federal Statistical Office. The cohort is recruited by postal mail. The procedure for cohort recruitment is shown in Fig. 1. Invitation letters include a study information document, an informed consent form, a contact information questionnaire (for obtaining participants’ contact details and for selection of the preferred language and means of communication) and a short screening questionnaire to assess study eligibility. Eligibility criteria for participating in the study are: living in Switzerland; speaking German, French or Italian; age ≥ 14 years; not suffering from cancer of the bowel, irritable bowel syndrome, Crohn’s disease, ulcerative colitis, cystic fibrosis, coeliac disease or another chronic illness with symptoms of diarrhoea or vomiting.

#### Data collection and management

**Fig. 2:**
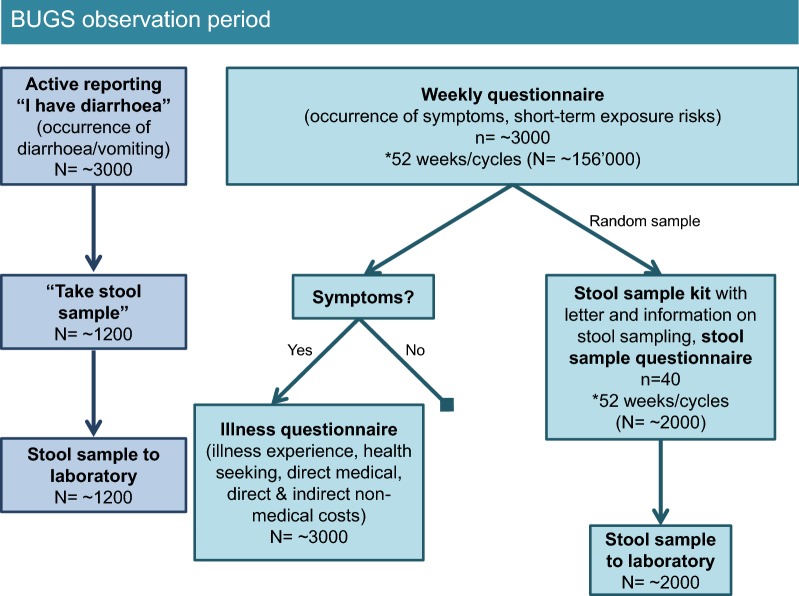
Flowchart of cohort observation period for the burden of gastroenteritis in Switzerland (BUGS) study

**Fig. 3:**
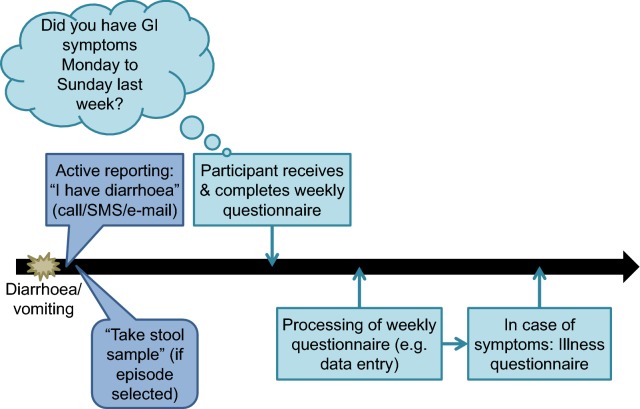
Timeline from occurrence of symptoms until sending of illness questionnaire for the burden of gastroenteritis in Switzerland (BUGS) study. GI, gastrointestinal

Upon return of the signed informed consent and the completed contact information and screening questionnaires, eligible study participants receive a baseline questionnaire, a stool sampling kit including an information and instruction sheet on stool sampling, and the first weekly questionnaire. The content of each questionnaire is summarised in Table 1. Participants will continuously receive the weekly questionnaire during 1 year (52 weeks; Fig. 2). Participants receive an additional questionnaire (“illness questionnaire”) in case of reporting gastrointestinal signs and symptoms. Furthermore, participants are advised to immediately report occurrence of diarrhoea and/or vomiting actively to the study team by phone, e-mail or SMS (during the day, including weekends) (Figs. 2 and 3). This “active reporting system” is used to select AG episodes for microbiological investigation based on a pre-defined algorithm considering moderate and severe cases of illness as defined by Riddle et al. [19]. In case the study subject’s AG episode is selected, the study team will advise him/her to use the stool sampling kit received at the beginning of the study and send a stool sample to the study laboratory as fast as possible. After sending in a stool sample, the participant’s stool sampling kit will be replaced. The list of diagnostic tests performed on stool samples from symptomatic participants is provided in Table 1.

**Table 1:** Overview and content of different questionnaires used and other data collection for the burden of gastroenteritis in Switzerland (BUGS) study

Data collection tool	Means of application	Frequency	Content
Contact information questionnaire	Paper-based	1× before enrolment	Address, e-mail, phone number, language, preferred means of communication (electronic questionnaire with link sent by e-mail [default] or paper-based questionnaire sent by postal mail)
Screening questionnaire	Paper-based	1× before enrolment	General demographic characteristics (age, sex), characteristics to assess eligibility; for those not willing to participate: reason for non-participation
Baseline questionnaire	Electronic and paper-based	1× at the beginning of the observation period	Baseline characteristics: detailed demographic characteristics, dietary habits, regular medication intake, chronic diseases, permanent and long-term exposure risks (e.g. occupational), general health seeking
Weekly questionnaire	Electronic and paper-based	Weekly (52×)	Occurrence of gastrointestinal signs and symptoms and short-term/transient risk exposures (e.g. food consumption, travel)
Illness questionnaire	Electronic and paper-based	After experiencing gastrointestinal signs and symptoms	Additional risk exposures (antibiotic use, hospital stay), disease determinants, health and help seeking, health care utilisation, (self-)medication (incl. antibiotic use), consultations, absence from work, ability to perform usual daily activities
Stool sample (symptomatic)	Sampling kit sent to participant at baseline; upon instruction by study personnel participant sends to study laboratory	Selected episodes of acute gastroenteritis	Stool sample investigated for: Bacteria: *Campylobacter* spp., *Salmonella* spp., *Shigella* spp., *Yersinia* spp. (all culture and PCR); *Clostridium difficile* (RDT and PCR); *Plesiomonas shigelloides*, *Vibrio* spp., EAEC, EPEC, ETEC, EHEC (all PCR) Viruses: adenovirus, astrovirus, norovirus, rotavirus, sapovirus (all PCR) Protozoa and parasites: *Cryptosporidium*, *Cyclospora cyetanensis*, *Entamoeba histolytica*, *Giardia lamblia* Selected samples (depending on risk profile): additionally for cestodes, trematodes, nematodes and protozoa
Stool sample (asymptomatic)	Sampling kit sent to participant; Participant sends to study laboratory	Max. 1× during observation period; random selection	Stool sample investigated for: Antibiotic resistance: ESBL, carbapenemase, fluoroquinolones; if carbapenemase-positive: MCR-1 Bacteria: *Campylobacter* spp., *Salmonella* spp., *Shigella*/EIEC, EHEC (all PCR)
Stool sample questionnaire (asymptomatic)	Electronic and paper-based	Max. 1× during observation period; random selection	Risk factors for carrying antibiotic-resistant bacteria, recent antibiotic consumption, visits to or stay in medical institutions, contact to animals and/or raw food

PCR, polymerase chain reaction; RDT, rapid diagnostic test; EAEC, enteroaggregative *Escherichia coli*; EPEC, enteropathogenic *E. coli*; ETEC, enterotoxigenic *E. coli*; EHEC, enterohaemorrhagic *E. coli*; ESBL, extended-spectrum beta-lactamase; MCR-1, mobilised colistin resistance-1; EIEC, enteroinvasive *E. coli*

Each week, a pre-defined number of randomly selected participants (see sample size calculation) will receive a stool sampling kit to send in a stool sample immediately. Those stool samples will be tested for selected pathogenic bacteria and presence of certain antibiotic resistances with the aim to assess respective prevalences in the asymptomatic population (Table 1). The stool sample will be discarded if the participant reported diarrhoea with or without vomiting in the 4 weeks preceding sampling. Sampling is conducted weekly throughout the observation period to account for potential seasonal differences in prevalence. Each participant is selected at most once for “asymptomatic sampling”.

All questionnaires apart from the contact information and screening questionnaires will be available in electronic- and paper-format. By default, participants will receive an e-mail containing a personalised link to the electronic questionnaires. However, during recruitment, they may opt for paper-based questionnaires sent by postal mail. LimeSurvey, an open source software set-up on a secured server at our institution (Swiss TPH), will be used for electronic data collection. The software allows completion of the questionnaire in a standard internet browser using desktop or laptop computers, tablets or smartphones.

Electronic questionnaires will be programmed to require an answer to ensure completeness of the data collected but will include an option “I do not want to answer” or “not applicable” (where appropriate) in order to prevent a forced-choice bias.

Paper questionnaires are entered by study personnel at the study centre using a LimeSurvey data entry mask which is slightly adapted from the one used by participants (e.g. containing an additional field “no answer given” for each mandatory question). A sample of 10% of paper questionnaires is entered twice for data quality control. Discordant results are checked against paper originals. A full double entry is conducted for the “contact information questionnaire” as correct address details are crucial for successfully contacting participants.

Quality control of data from study laboratories (stool sample results) will be with the diagnostic laboratories, operating according to their standard operating procedures in their daily routine. The study team will only check plausibility of the data and values.

Electronic data are stored on secured network drives accessible only by the study team. Data on the network drive is backed-up regularly, according to institutional policy. Electronic data is stored in original file formats and in comma-separated values (csv) format, where appropriate, to ensure long-term accessibility.

#### Sample size calculation

We calculated the minimal sample size based on different parameter assumptions for comparisons between two distinct groups and for within-subject comparisons between two distinct periods. Underlying formulas and results are provided in Additional file 1.

A cohort size of 3000 individuals is envisaged to allow analysis of secondary outcomes and comparisons between groups and to assert enough power in case assumptions were too optimistic based on the different sample size calculations. We plan to start the observation period with a cohort size of 3500 individuals in order to achieve an average cohort size of 3000 after withdrawals and loss to follow-up during the 52 weeks study period. Assuming a participation rate of 25% (including loss of people not meeting the eligibility criteria) and a loss to follow-up of 20%, 15,000 people will be contacted initially. For planning purposes, we assume an average cohort size of 3000 throughout the 1-year observation period.

The sample size (n) needed to reach a specific relative precision (ε) for the microbiological outcomes in asymptomatic participants (prevalence of antibiotic resistances and selected pathogenic bacteria) was calculated using the following formula (based on [20]):

$$n = \frac{{1.96^{2} *\left( {1 - P} \right)}}{{\varepsilon^{2} *P}}$$

For the prevalence of ESBL, a relative precision of 20% is envisaged at a 95% confidence level. Assuming a prevalence (P) of 5–6% (based on [21, 22]), a sample size of 1505–1825 is needed. Considering that the previous prevalence estimates are from groups of potentially higher prevalence than the general population (staff members of meat-processing companies with likely higher exposure risk; and primary care patients), we plan to investigate 2000 samples. Hence, every week 40 subjects are randomly selected. Given the very low prevalence of Carbapenemase-resistance (0.1%; personal communication) and mcr-1 (not found in 1000 samples; personal communication) it is questionable whether these resistances will be found at all in our cohort. The prevalence of fluoroquinolone-resistance in *E. coli* has not yet been investigated in the asymptomatic Swiss population. Fluoroquinolone resistance prevalence in *E. coli* is at around 20% based on resistance data generated during routine medical care [23]. Hence, using the same sample size as for ESBL (N = 2000) should allow for an estimate with a relative precision of at least 20% even if the prevalence in the general Swiss population is somewhat lower than 20%.

The presence of pathogenic bacteria (EHEC, *Campylobacter* spp., *Salmonella* spp., and *Shigella* spp.) can only be investigated in 1600 samples of asymptomatic participants (of 2000 collected) due to financial constraints. For this investigation, the main interest is on EHEC prevalence in asymptomatic people. Considering that this prevalence is expected at around 7–10% (personal communication) a sample size of 1600 will still allow for a relative precision of 15–18%.

### Approach to analysis

#### Definition of AG disease episode

The primary outcome of our study is presence or absence of an AG episode and the incidence of AG in the general population. For this purpose, an episode of AG is classified according to a modified version of the definition suggested by Majowicz et al. [24]: a case of AG is an individual with ≥ 3 loose stools in 24 h, with or without vomiting, but excluding those (a) with cancer of the bowel, irritable bowel syndrome, Crohn’s disease, ulcerative colitis, cystic fibrosis, coeliac disease, or another chronic illness with symptoms of diarrhoea, or (b) who report their symptoms were due to drugs, alcohol, or pregnancy. An episode is defined to begin on the first day and end on the last day of reported diarrhoea and/or vomiting, followed by a diarrhoea- and vomiting-free period of 3 days [25].

#### Statistical analysis

Data cleaning and analysis will be conducted with the statistical software Stata and/or R. All steps performed to clean and analyse the data are documented in scripts (R) or do-files (Stata).

The cohort will be characterised in terms of demographic characteristics, health status, dietary habits and permanent or long-term risk exposures as reported at baseline. Similarly, cohort participants lost to follow-up will be compared to those remaining in the cohort.

The primary outcome defined at the level of person-week is presence or absence of an AG episode. Risk factors for experiencing an episode of AG will be determined using multivariable mixed logistic regression analyses with the individual included as a random effect. A basic multivariable model including the biologically most plausible variables, region and season will be defined a priori. Generalised estimating equation (GEE) models will be considered in case of convergence issues of the mixed logistic regression models.

Secondary analyses will include calculating the incidence of AG as the number of AG episodes per person-year under observation and the incidence of gastrointestinal signs and symptoms (including episodes not fulfilling the case definition of AG). Furthermore, transient risk factors for experiencing an episode of AG will be explored in uni- and multivariable mixed logistic regression models, including the individual as random effect. Exposure information from the week preceding the AG episode will be used. Determinants for presenting to the health system (pharmacies, primary care, specialists or hospitals) in case of AG are also investigated, again using uni- and multivariable mixed logistic regression models (with the individual as random effect). Predictor variables include signs and symptoms, and perceived severity of AG, demographic characteristics (age, sex, occupational status), co-morbidity, and type of health insurance. Additionally, the socio-economic impact such as absence from work, inability to perform usual daily activities and the need for care by family members or friends related to AG disease episodes will be described.

Results from stool sample investigations from symptomatic participants will be used to estimate pathogen-specific incidence rates of AG.

Finally, the prevalences of bacteria harbouring ESBL-, carbapenem- and fluoroquinolone-resistance, and of EHEC, *Campylobacter*, *Salmonella* and *Shigella* in the asymptomatic population are calculated.

### Ethical considerations

Health-related personal data and stool samples from participants are collected for this study. Therefore, the study is subject to the Federal Act on Research on Human Beings and requires ethical approval. Approval will be sought from the responsible local ethical committee(s) for a non-clinical trial study with minimal risks. All participants will be asked for written informed consent before enrolment. The study will be conducted according to the principles of Good Epidemiological Practice [26] and the Declaration of Helsinki [27].

Participants will be advised to seek health care as they would do without participating in this study. Similarly, it will be emphasised that investigation of stool samples in the framework of this study does not replace stool sample investigation potentially initiated by their health care provider. Results of stool investigations of symptomatic participants conducted as part of this study are obtained with a time delay due to study logistics. Hence, microbiological results will be known to the study team too late to affect treatment considering the short disease duration of most AG episodes and, therefore, will not be communicated to participants or their physicians.

Participants are informed if antibiotic resistances are identified in their stool samples during an asymptomatic period and advised to inform their physician in case of illness.

### Operational issues

#### Data confidentiality and personalisation

Ideally, questionnaires should be anonymous as soon as they contain health-related personal information. However, we must be able to link the different questionnaires completed by each study participant. To minimise the risk that unauthorised people are able to link the participants’ names to their questionnaire data, we plan to implement several measures: each participant is given a person identification code (“person ID”) as well as a questionnaire identification code (“questionnaire ID”). The person ID is used in all files/data sets containing personal information. The questionnaire ID is used in all files/data sets containing information obtained from questionnaires or from laboratory testing. The key file, linking the person ID and the questionnaire ID will be password protected and stored separately from the other data sets. Access to this key file will be limited to the principal investigator, the study coordinator and an additional person (substitute of the study coordinator). For paper-based questionnaires, both codes (person ID and questionnaire ID) are printed on the empty questionnaires. However, as soon as receipt of the completed questionnaires is registered in the study centre, the person ID will be removed (cut off). In order to avoid that both codes are easily visible and recognised as such by unauthorised persons, one of the codes (the person ID) is printed in directly readable format (Arabic letters and numerals) and the other code is printed as a QR- or bar-code. Also, the two codes are not labelled with “person ID” or “questionnaire ID”. For electronic questionnaires only the “questionnaire ID” will be used and no names are stored in LimeSurvey (where questionnaire data is entered and stored during the entire data collection phase). In order to complete the questionnaire, participants will receive an e-mail including a link to the questionnaire containing the questionnaire ID.

#### Active reporting system needed

For investigating the aetiology of AG, stool samples need to be obtained quickly after disease onset as AG is usually a short, self-limiting disease. Therefore, the information on signs and symptoms obtained through the weekly questionnaire is too much delayed to be the basis for selection of episodes for aetiological investigation (especially in those completing the paper-based questionnaires; Fig. 3). We expect reporting delays of 1–13 days in those completing electronic questionnaires and of at least 3–10 days (best case scenario) for paper-based questionnaires which will severely impact the likelihood to detect pathogens. To address this limitation, we plan to set up an “active reporting system” for participants (see “Data collection and management”). These parallel ways of collecting data on diarrhoea and vomiting have advantages and disadvantages: on the one hand, we can compare reporting completeness of the two methods. On the other hand, participants are required to actively think of the study when experiencing signs and symptoms, and have to report their symptoms twice—potentially increasing reporting fatigue and reporting bias due to sensitisation. Further, the active reporting system requires that study personnel being able to communicate in all three study languages is on call every day, including weekends and public holidays.

## Limitations

Our study will be subject to limitations. Participation bias is likely to occur. Considering the rather long observation period (1 year with weekly follow-ups), a certain amount of participants will be lost to follow-up or withdraw from the study; these participants might differ from those completing the entire follow-up period. Similarly, reporting fatigue might occur, especially in those experiencing more than one episode of AG. However, all those biases and limitations are unavoidable in cohort studies and their mitigation is difficult. To address reporting fatigue, we consider establishing a project newsletter to give participants feedback about interim results of the study and additional information related to the topic to highlight the importance of their contribution to research. Inverse probability weighting will be considered if characteristics of our final cohort differ from those of the general population.

Furthermore, compliance in actively reporting signs and symptoms as well as in providing stool samples is crucial. Therefore, its importance will be emphasised repeatedly to study participants. We suspect that compliance might be associated with the (perceived) severity of the disease episode. However, we can only try to assess this bias at the end of the study by comparing data from active reporting/stool sampling and data from weekly questionnaires.

Recall bias and telescoping have been described as major challenges in studies on AG. We believe that this bias might not be a notable problem in our study considering the rather short recall period of 1 week.

Participants travelling will still receive the electronic questionnaire but those completing paper-based questionnaires will be able to complete the questionnaire only once they returned. This might lead to different compliance and recall bias in those two groups. On the other hand, it provides an opportunity to assess potential differences in results between the two methods.

## Additional file

**Additional file 1.** Sample size calculations and underlying formulas for the BUGS study. Sample size calculations for the burden of gastroenteritis in Switzerland (BUGS) study based on different parameter assumptions, including derivation of underlying formulas.

## Abbreviations

AG: acute gastroenteritis
BUGS: burden of gastroenteritis in Switzerland [study]
EAEC: enteroaggregative *Escherichia coli*
EHEC: enterohaemorrhagic *Escherichia coli*
EIEC: enteroinvasive *Escherichia coli*
EPEC: enteropathogenic *Escherichia coli*
ESBL: extended-spectrum beta-lactamase
ETEC: enterotoxigenic *Escherichia coli*
FOPH: Federal Office of Public Health
mcr: mobilised colistin resistance
NNSID: National Notification System for Infectious Diseases
PCR: polymerase chain reaction
RDT: rapid diagnostic test
