# Supplementary material for: The burden of gastroenteritis in Switzerland (BUGS) study: a research proposal for a 1-year, prospective cohort study
Source: BMC Res Notes. 2018 Nov 16;11:816. doi: 10.1186/s13104-018-3916-2 (PMC6240284; doi:10.1186/s13104-018-3916-2)
Supplement: Supplementary file 1 — Additional file 1. Sample size calculations and underlying formulas for the BUGS study. Sample size calculations for the burden of gastroenteritis in Switzerland (BUGS) study based on different parameter assumptions, including derivation of underlying formulas. [file 13104_2018_3916_MOESM1_ESM.pdf]

Additional file 1

The burden of gastroenteritis in Switzerland (BUGS) study: a research proposal for a one-year, prospective cohort study

Claudia Schmutz[1,2], Daniel Mäusezahl[1,2]*

[1] Swiss Tropical and Public Health Institute, Basel, Switzerland

[2] University of Basel, Basel, Switzerland

* Corresponding author (daniel.maeusezahl@swisstph.ch)

Sample size calculation for the burden of gastroenteritis in Switzerland (BUGS) study based on different parameter assumptions, including derivation of underlying formulas.

Comparison between two distinct groups (exposed and unexposed)

Power calculations were based on Hayes and Bennett [1], with adjustment for the situation of unequal group sizes.

Let λ = average event rate among exposed persons (expressed as number of events per person year)
$d\lambda$ = hypothesized difference in average event rate between exposed and unexposed persons
λ_1 = average event rate among exposed persons
λ_0 = average event rate among exposed persons
q = proportion of exposed person

Then,

$\lambda_1 = \lambda + (1-q) \cdot d\lambda$ and $\lambda_0 = \lambda - q \cdot d\lambda$.

The corresponding weekly event rates are $\lambda_1{}' = \frac{\lambda_1}{52}, \lambda_0{}' = \frac{\lambda_0}{52}$.

Let y = average number of weeks of observation per subject.

Then, formula (2) of Hayes and Bennett [1] was adapted and slightly simplified to provide the minimal total number N of subjects required as

$$N = \left(1.96 + z_{1-\beta}\right)^2 \cdot \left[\left(\frac{\lambda_1{}'}{q} + \frac{\lambda_0{}'}{1-q}\right)/y + CV^2 \cdot \left(\frac{\lambda_1{}'^2}{q} + \frac{\lambda_0{}'^2}{1-q}\right)\right] \;/\; (\lambda_1{}' - \lambda_0{}')^2 ,$$

where β denotes the accepted type II-error probability. In case of q = 0.5, N equals 2·(c – 1), where c = number of clusters per group according to formula (2) of Hayes and Bennett [1].

Table 1 Sample size calculation for the burden of gastroenteritis in Switzerland (BUGS) study for a comparison of the mean number of events per person and year between two distinct groups (exposed and unexposed) based on different parameter assumptions

**Parameters specified / assumptions**						**Comparison of mean number of events per person and year between two distinct groups (exposed and unexposed)**
Mean number of events per person and year	Difference in mean number of events per person and year between exposed and unexposed	Power	Proportion exposed	ICC	Average number of weeks observed	Calculated sample size needed
1	0.3	0.9	0.2	0.2	40	1162 (n_1=929, n_2=233)
0.75	0.3	0.9	0.2	0.2	40	909 (n_1=727, n_2=182)
0.75	0.3	0.9	0.2	0.2	26	1384 (n_1=1107, n_2=277)
0.75	0.3	0.9	0.2	0.2	52	705 (n_1=564, n_2=141)
0.75	0.2	0.9	0.2	0.2	40	1908 (n_1=1526, n_2=382)
0.75	0.2	0.8	0.2	0.2	40	1425 (n_1=1140, n_2=285)
0.75	0.1	0.8	0.2	0.2	40	**5295 (n_1=4236, n_2=1059)**
0.75	0.2	0.9	0.2	0.5	40	2172 (n_1=1737, n_2=435)
0.75	0.2	0.9	0.2	0.5	26	**3172 (n_1=2537, n_2=635)**
0.75	0.2	0.8	0.2	0.5	26	2369 (n_1=1895, n_2=474)
0.75	0.3	0.9	0.1	0.2	40	1722 (n_1=1549, n_2=173)
0.75	0.2	0.9	0.1	0.2	40	**3552 (n_1=3196, n_2=356)**
0.75	0.2	0.8	0.1	0.2	40	2653 (n_1=2387, n_2=266)
0.5	0.3	0.9	0.2	0.2	26	1008 (n_1=806, n_2=202)
0.5	0.3	0.9	0.2	0.2	40	660 (n_1=528, n_2=132)
0.5	0.3	0.8	0.2	0.2	26	753 (n_1=602, n_2=151)
0.5	0.3	0.9	0.2	0.5	40	733 (n_1=586, n_2=147)
0.5	0.2	0.9	0.2	0.5	40	1484 (n_1=1187, n_2=297)
0.5	0.1	0.9	0.2	0.5	40	**5299 (n_1=4239, n_2=1060)**
0.5	0.1	0.8	0.2	0.2	40	**3634 (n_1=2907, n_2=727)**

ICC = intraclass correlation coefficient
bold: Calculated minimal sample size exceeds envisaged cohort size of 3000 individuals

Within-subject comparison between two distinct periods (high and low risk period): Longitudinal comparison of rates

Let $X_{ij}^{(1)}$ = number of events in subject i during unit interval j (e.g., week j) in low risk period
Let $X_{ij}^{(2)}$ = number of events in subject i during unit interval j (e.g., week j) in high risk period

Assumptions:

(1) $X_{ij}^{(1)} = \text{Pois}(\lambda_1 + d\lambda_{i0} + d\lambda_{i1})$, $j = 1, \ldots, m_1$
(2) $X_{ij}^{(2)} = \text{Pois}(\lambda_2 + d\lambda_{i0} + d\lambda_{i2} + d\lambda_{i3})$, $j = m_1 + 1, \ldots, m_1 + m_2$

where

λ_1 and λ_2 denote the average rates in the low and high risk period, respectively,
$d\lambda_{i0}$ denotes a subject-specific random effect with $E(d\lambda_{i0}) = 0$ across both periods,
$d\lambda_{i1}$ and $d\lambda_{i2}$ denote subject-specific random period effects with $E(d\lambda_{i1}) = E(d\lambda_{i2}) = 0$ and $E(d\lambda_{i1}^2) = E(d\lambda_{i2}^2)$,
and $d\lambda_{i3}$ denotes an additional subject-specific random effect in the high risk period with $E(d\lambda_{i3}) = 0$.

All random effects are assumed to be mutually independent.

We define:

$$U_i = \frac{1}{m_2}\sum_{j=m1+1}^{m1+m2} X_{ij}{}^{(2)} - \frac{1}{m_1}\sum_{j=1}^{m1} X_{ij}{}^{(1)}$$

The variance of U_i conditional on fixed values of the random effects then becomes:

$$Var(U_i|random\,effects) = \frac{1}{m_2}(\lambda_2 + d\lambda_{i0} + d\lambda_{i2} + d\lambda_{i3}) + \frac{1}{m_1}(\lambda_1 + d\lambda_{i0} + d\lambda_{i1})$$

giving

$$E[Var(U_i|random\,effects)] = \frac{1}{m_2}\lambda_2 + \frac{1}{m_1}\lambda_1 \qquad (1)$$

Moreover,

$$E(U_i|random\,effects) = (\lambda_2 + d\lambda_{i0} + d\lambda_{i2} + d\lambda_{i3}) - (\lambda_1 + d\lambda_{i0} + d\lambda_{i1})$$
$$= (\lambda_2 - \lambda_1) + (d\lambda_{i2} - d\lambda_{i1}) + d\lambda_{i3}$$

implying that

$$E[E(U_i|random\,effects)^2] = (\lambda_2 - \lambda_1)^2 + \mathrm{Var}(d\lambda_{i2}) + Var(d\lambda_{i1}) + Var(d\lambda_{i3})$$

and

$$Var[E(U_i|random\,effects)] = \mathrm{Var}(d\lambda_{i2}) + Var(d\lambda_{i1}) + Var(d\lambda_{i3}) \qquad (2)$$

For the variance of U_i, which is the sum of (1) and (2), we thus obtain

$$Var(U_i) = \frac{1}{m_2}\lambda_2 + \frac{1}{m_1}\lambda_1 + 2\cdot \mathrm{Var}(d\lambda_{i1}) + Var(d\lambda_{i3}) \qquad (3)$$

If we assume that $Var(d\lambda_{i1}) = Var(d\lambda_{i2}) = Var(d\lambda_{i0})$ and relate $Var(d\lambda_{i0})$ to λ_1 through

$$Var(d\lambda_{i0}) = (\lambda_1 \cdot CV_1)^2,$$

where CV_1 is the coefficient of variation of $\lambda_1 + d\lambda_{i0}$, and $Var(d\lambda_{i3})$ to $|\lambda_2 - \lambda_1|$ through

$$Var(d\lambda_{i3}) = (|\lambda_2 - \lambda_1| \cdot CV_2)^2\ ,$$

where CV_2 is the coefficient of variation of $|\lambda_2 - \lambda_1| + d\lambda_{i3}$,

then we finally obtain

$$Var(U_i) = \frac{1}{m_2}\lambda_2 + \frac{1}{m_1}\lambda_1 + 2\cdot(\lambda_1 \cdot CV_1)^2 + (|\lambda_2 - \lambda_1| \cdot CV_2)^2. \qquad (4)$$

In our power calculations we chose $CV_1 = CV_2 = 0.25$. If all random effects were normally distributed, this would imply that $d\lambda_{i1},\ d\lambda_{i2} \in (-0.5\cdot\lambda_1, 0.5\cdot\lambda_1)$ and $d\lambda_{i3} \in (-0.5\cdot|\lambda_2 - \lambda_1|, 0.5\cdot|\lambda_2 - \lambda_1|)$, each with a probability of 95%.

Table 2 Sample size and power calculation for the burden of gastroenteritis in Switzerland (BUGS) study for a comparison of the mean number of events per person and year between two distinct periods (high and low risk period) based on different parameter assumptions

**Parameters specified / assumptions**						**Comparison of mean number of events per person and year between two distinct periods**
Mean number of events per person and year	Difference in mean number of events per person and year between high and low risk period	Power	Proportion observed weeks at high risk	Individual coefficient of variation	Average number of weeks observed	Calculated sample size needed
1	0.3	0.9	0.2	0.25	40	1133
0.75	0.3	0.9	0.2	0.25	40	890
0.75	0.3	0.9	0.2	0.25	26	1365
0.75	0.3	0.9	0.2	0.25	52	687
0.75	0.2	0.9	0.2	0.25	40	1875
0.75	0.2	0.8	0.2	0.25	40	1400
0.75	0.1	0.8	0.2	0.25	40	**5219**
0.75	0.2	0.9	0.2	0.5	40	1926
0.75	0.2	0.9	0.2	0.5	26	2926
0.75	0.2	0.8	0.2	0.5	26	2186
0.75	0.3	0.9	0.1	0.25	40	1678
0.75	0.2	0.9	0.1	0.25	40	**3472**
0.75	0.2	0.8	0.1	0.25	40	2593
0.5	0.3	0.9	0.2	0.25	26	996
0.5	0.3	0.9	0.2	0.25	40	649
0.5	0.3	0.8	0.2	0.25	26	744
0.5	0.3	0.9	0.2	0.5	40	659
0.5	0.2	0.9	0.2	0.5	40	1354
0.5	0.1	0.9	0.2	0.5	40	**4905**
0.5	0.1	0.8	0.2	0.25	40	**3595**

bold: Calculated minimal sample size exceeds envisaged cohort size of 3000 individuals

References

1. Hayes RJ, Bennett S: **Simple sample size calculation for cluster-randomized trials.** *Int J Epidemiol* 1999, **28:**319-326.
